# Preoperative Screening CT and PET/CT Scanning for Acral Melanoma: Is it Necessary?

**DOI:** 10.3390/jcm10040811

**Published:** 2021-02-17

**Authors:** Taketoshi Ide, Takamichi Ito, Maiko Wada-Ohno, Masutaka Furue

**Affiliations:** Department of Dermatology, Graduate School of Medical Sciences, Kyushu University, 3-1-1 Maidashi, Higashi-ku, Fukuoka 812-8582, Japan; takamiti@dermatol.med.kyushu-u.ac.jp (T.I.); maiko1027wada@gmail.com (M.W.-O.); furue@dermatol.med.kyushu-u.ac.jp (M.F.)

**Keywords:** acral melanoma, prognosis, sentinel lymph node, metastasis, computed tomography, positron emission tomography

## Abstract

The efficacy of preoperative imaging for acral melanoma (AM) has not been fully evaluated. We examined the accuracy of imaging modalities in the detection of nodal and distant metastases in patients with AM. A retrospective review of 109 patients with AM was performed. All patients had no clinical signs suggestive of distant metastases, and underwent preoperative screening computed tomography (CT) and positron emission tomography (PET)/CT scans. Of 100 patients without lymphadenopathy, 17 patients were suspected of having nodal metastasis in CT and PET/CT, but only two of them were confirmed on histopathological analysis. On the other hand, 12 out of 83 negatively imaged patients showed histopathological signs of nodal metastasis; thus, the sensitivity and specificity of nodal detection were 14.3% and 82.6%, respectively. Regard to the detection of distant metastases, four patients were suspected of having metastasis, but this was later ruled out. The remaining 96 negatively imaged patients were confirmed to have no metastasis at the time of CT and PET/CT by the follow-up. In contrast, distant metastases were found by CT and PET/CT in four of nine patients (44.4%) with lymphadenopathy. Routine preoperative CT and PET/CT for AM patients without lymphadenopathy may not be warranted because of low sensitivity and specificity, but it can be considered for those with lymphadenopathy.

## 1. Introduction

Malignant melanoma is an aggressive malignant tumor derived from melanocytes, and its incidence is increasing worldwide [1,2]. Melanoma accounts for a small percentage of skin cancers, but accounts for the majority of deaths from such cancers [3]. Although melanoma can occur all over the body, it occurs most frequently on the skin. Cutaneous malignant melanomas are classified into several subtypes based on anatomical site and degree of ultraviolet light exposure. The acral melanoma (AM) subtype arises on the glabrous skin of the palms, soles, and nail apparatus [4]. Among melanomas, AM represents a peculiar subtype since the genetic background is different from those of other subtypes and the ultraviolet irradiation is less associated with its pathogenesis [5]. Owing to its rarity in Caucasians, AM has not been intensively investigated [6].

From the first introduction of sentinel lymph node (SLN) biopsy, the procedure has been widely accepted and has become the standard approach for staging melanoma. SLN biopsy is conducted to detect occult metastases within regional lymph nodes when patients do not show clinically detectable metastases [7]. Imaging modalities such as computed tomography (CT) and positron emission tomography (PET)/CT may be used before SLN biopsy to detect that patients are eligible for the procedure (not having lymph node or distant metastasis). CT and PET/CT may also be applied to search for synchronous distant metastases in patients with regional lymphadenopathy. Numerous studies have been conducted on the usefulness of imaging modalities for screening purposes and revealed various levels of sensitivity and specificity depending on the time points in the clinical course and the site of metastasis [8,9,10,11,12]. A Cochrane review calculated low sensitivity (10.2%, 95% confidence interval (CI) 4.31% to 22.3%) and high specificity (96.5%, 95% CI 87.1% to 99.1%) of detecting nodal metastasis by PET/CT before SLN biopsy [3]. For whole-body imaging via PET/CT for primary staging, limited test accuracy data are available; only six studies that explored primary staging following a confirmed diagnosis of melanoma were eligible and included in a meta-analysis, showing low sensitivity and specificity (sensitivity ranging from 30% to 47% and specificity from 73% to 88%) [3]. As such, current guidelines do not recommend the routine use of CT or PET/CT for patients without lymphadenopathy [13,14,15]. However, data regarding the efficacy of preoperative imaging for staging AM are still lacking. Contrary to the case in Western countries, AM is the dominant subtype of melanoma in Japan [16]. Owing to easy access to imaging modalities, preoperative CT and PET/CT have been routinely performed in Japan. Here, we summarize our 17-year experience of treating patients with AM and evaluate the efficacy of CT and PET/CT in the context of preoperative staging. In this study, we examined the accuracy of CT and PET/CT in the detection of nodal metastasis and distant metastasis before SLN biopsy and of distant metastasis in patients with lymphadenopathy.

## 2. Materials and Methods

### 2.1. Ethics Statement

We conducted this investigation in accordance with the concepts enshrined in the Declaration of Helsinki. This study was approved by the Institutional Ethics Committee of Kyushu University (30-363; 27 November 2018).

### 2.2. Patients

A retrospective database of 168 acral melanoma cases was used. All patients were Japanese and diagnosed as having acral melanoma (histopathologically, acral lentiginous melanoma) between 2001 and 2018 at Kyushu University Hospital. A total of 59 patients were excluded from this study for the following reasons: 29 patients did not undergo preoperative imaging, 25 had a lack of detailed information on the tumor, three had known distant metastasis, and two had received treatments at other hospitals. The remaining 109 patients had no clinical signs suggestive of distant metastasis and underwent preoperative screening CT with or without PET/CT scans. Of these 109 patients, nine had clinically enlarged regional lymph nodes, which were also detected on imaging. We analyzed all of these 109 patients to elucidate the clinical significance of preoperative imaging modalities. Twenty patients underwent CT alone and the remaining 89 patients underwent CT and PET/CT.

### 2.3. Treatment and Follow-up

Patients were treated in accordance with the National Comprehensive Cancer Network (NCCN) melanoma guidelines [13]. After the preoperative screening for metastasis, patients underwent complete resection of the primary tumor together with SLN biopsy or completion lymph node dissection (CLND). SLN biopsy was performed for patients without lymphadenopathy and CLND was performed for those with lymphadenopathy. SLN biopsy was performed in accordance with the standard procedure as reported previously [17]. Preoperative lymphoscintigraphy with Tc-99m phytate, intraoperative lymphatic mapping with patent blue violet, and radioactivity measured with a gamma probe were used for the detection of SLN. Each SLN was evaluated by the combination of hematoxylin and eosin (HE) staining and immunohistochemical staining (S100 protein, Melan A, HMB45, etc.). Patients with SLN metastases subsequently received CLND. Histopathological data of lymph nodes were available for 90 patients (85.7%) and not for the other 15 patients (14.3%) who did not undergo either lymph node biopsy (including SLN biopsy) or CLND. No patient had received neoadjuvant therapy with immunotherapy, targeted therapy, or other conventional chemotherapies before the surgery. Follow-up with postoperative screening imaging were performed every 3–12 months depending on the tumor stage.

### 2.4. Detection of Lymph Node and Distant Metastasis by Imaging Modalities

We analyzed whether imaging modalities detected occult lymph node metastases. Any finding suggestive of metastases, in either CT or PET/CT, was considered as positive. The accuracy of the imaging was then evaluated using the histopathological findings. Lymph node tissues were obtained by lymph node biopsy (including sentinel lymph node biopsy) or CLND; core needle biopsy or fine needle aspiration cytology was not performed.

We also analyzed whether imaging modalities detected occult distant metastases. Any finding suggestive of metastases, in either CT or PET/CT, was considered as positive. The findings in these imaging modalities were evaluated by follow-up imaging modalities or the clinical course of the patients. Patients with a follow-up period of less than 3 months were excluded from this study since the initial findings of the imaging modalities could not be confirmed. Cases with negative findings at the initial imaging were regarded as false-negative when detectable metastases emerged within 3 months and as newly emerging metastasis when they arose 3 months after the initial imaging.

## 3. Results

### 3.1. Patient Data

Table 1 shows background data of all 109 patients. All patients were Japanese, 46 patients (42.2%) were male and 63 (57.8%) female, and the median age was 66.3 years (range 16–90). The primary tumor site was predominantly the feet (*n* = 64, 58.7%), followed by the hands (*n* = 21, 19.3%), toenails (*n* = 14, 12.8%), and fingernails (*n* = 10, 9.2%). Regarding Breslow thickness, 22 patients (20.2%) were categorized as Tis, 25 (22.9%) as T1, 14 (12.8%) as T2, 15 (13.8%) as T3, and 33 (30.3%) as T4. Ulceration of primary tumor was observed in 33 tumors (30.3%). Regional lymphadenopathy was noted in nine patients (8.3%). As preoperative screening for occult metastases, 20 patients (18.3%) underwent a CT scan alone, while the remaining 89 (81.7%) underwent both CT and PET/CT.

### 3.2. Detection of Lymph Node and Distant Metastasis

**Figure 1:**
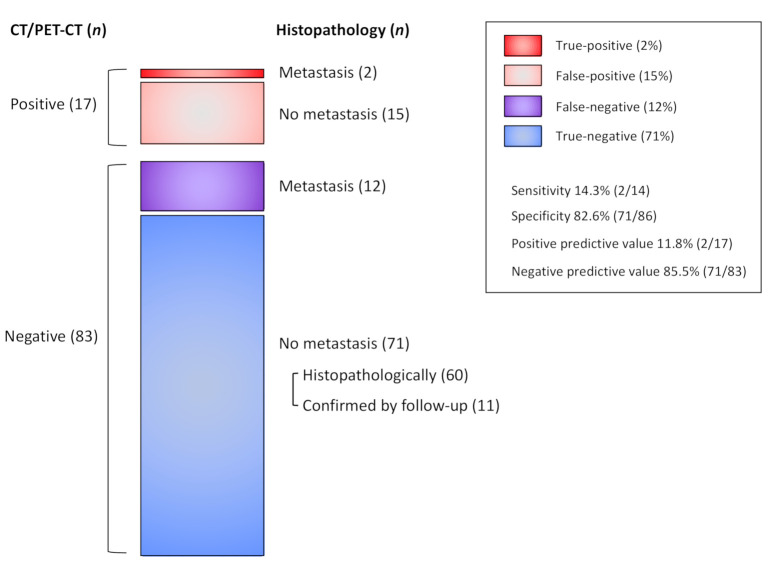
Nodal status of all patients. CT, computed tomography; PET, positron emission tomography.

Table 2 summarizes the detailed data of CT and PET/CT regarding the detection for lymph node and distant metastasis. Figure 1 shows a summary of lymph node metastasis.

For the detection of lymph node metastasis, 17 patients received a CT scan alone and 83 patients were assessed by the combination of CT and PET/CT. Two patients in the CT alone group were suspected of having metastatic lymph nodes; however, only one of these patients had histopathologically proven positive lymph nodes and no metastatic lymph nodes were noted by histopathological analysis in the other patient. The positive predictive value of preoperative imaging was thus 50.0%. In the CT and PET/CT group, lymph node metastases were suspected in 15 patients (18.1%) and all patients had received subsequent histopathological assessment. Only one patient had histopathologically positive lymph nodes and the other 14 patients appeared to have no lymph node metastasis after histopathological evaluation. The positive predictive value was low at 6.7%. Overall, only two patients had actual lymph node metastasis among 17 patients with signs of metastasis in imaging modalities, with a positive predictive value of 11.8% (2/17). Among the 83 patients without findings of nodal metastasis on imaging, 72 had undergone histological examination of regional nodes and 12 had been proven to have histopathologically detectable metastases, 60 had been histopathologically proven to have no metastases, and 11 had been proven to have no metastases by follow-up. Therefore, the sensitivity was 14.3% (2/14), the specificity was 82.6% (71/86), and the false negative rate was 14.5% (12/83). 

For the detection of distant metastasis, the number of suspicious cases was low, with one patient (5.9%) in the CT alone group and three patients (3.6%) in the CT and PET/CT group. In all the patients with signs suggestive of distant metastasis, metastasis was then ruled out by careful follow-up; suspicious lesions in the three patients disappeared without any systemic therapy at the follow-up imaging and the lesion in the other patient was a coincidental lung adenocarcinoma. The positive predictive value was thus 0%.

### 3.3. Breslow Thickness (T category) and Metastasis in Patients without Lymphadenopathy

Since Breslow thickness is a strong predictor of metastasis in acral melanoma [18], we next investigated the correlation between Breslow thickness and the positivity of imaging modalities (Table 3). For lymph node metastasis, suspicious signs were observed in four patients (18.2%) in Tis, four patients (16.7%) in T1, two patients (14.3%) in T2, two patients (15.4%) in T3, and five patients (18.5%) in T4, the total was 100 patients. Interestingly, no patients in Tis or in T1–3 had lymph node metastasis (all cases were false-positive), whereas two patients in T4 had histopathologically detected lymph node metastases with a positive predictive value of 40.0%.

Distant metastases were found in two patients (14.3%) in T2, one patient (7.7%) in T3, and one patient (3.7%) in T4. As already mentioned, none of the patients had distant metastasis of melanoma; thus, the positive predictive value was 0% in these T categories. Among patients with Tis or T1 acral melanoma, there were no signs of metastasis in CT and PET/CT.

### 3.4. Detection of Distant Metastasis in Patients with Lymphadenopathy

Table 4 summarizes the patients with lymphadenopathy at the time of initial diagnosis. Distant metastases were detected in 4 of 9 patients (44.4%): 2 in paraaortic lymph nodes, 1 in the liver, and 1 in the lung.

## 4. Discussion

Treatment strategy for melanoma depends on the detection of synchronous metastases before the surgery for primary melanoma will be changed. Accurate staging of melanoma is imperative to ensure patients to be directed to the most appropriate and effective treatment [3]. Routine preoperative imaging has been performed. However, such uniform imaging for all patients could provide inaccurate information, constitute a significant economic burden, and delay therapeutic intervention. In the current study, we addressed the issue of the efficacy of preoperative CT and PET/CT in patients with AM.

Interestingly, nodal or distant metastasis was extremely rare in AM patients without lymphadenopathy (two patients (2.0%) and no patients (0%), respectively). Nodal or distant metastasis was suspected by CT and PET/CT in 17 patients (17.0%) and four patients (4.0%), resulting in sensitivity of 14.3% and specificity of 82.6% for nodal detection and specificity of 96.0% (sensitivity could not be calculated) for distant metastasis. From another perspective, these results indicate low positive predictive values of 11.8% for nodal detection and 0% for distant metastasis detection, suggesting limited efficacy of routine preoperative imaging for patients without lymphadenopathy because of the extremely low possibility of synchronous metastases.

When looking at patients without lymphadenopathy more specifically regarding Breslow thickness (T category), true positive lymph nodes were noted only in patients with T4 (Breslow thickness > 4 mm) and none in those with thinner (≤ 4 mm) AM. Distant metastasis was not observed regardless of T category. These results may indicate that preoperative CT and PET/CT for staging is not necessary for AM patients without lymphadenopathy and SLN biopsy should be preferred for the detection of occult nodal metastasis, which accords well with the recommendations of the current guidelines [13,14,15]. Furthermore, considering the high rate of false-positivity (88.2%) for nodal detection, immediate CLND upon the suspicion of metastasis in imaging tests may not be preferred; CLND should be performed at least after confirming nodal metastasis with SLN biopsy, although immediate CLND for SLN-positive AM is still under debate [7].

Meanwhile, a non-negligible proportion of patients (4/9 = 44.4%) had synchronous distant metastases detected by CT and PET/CT among patients with lymphadenopathy, suggesting the necessity of preoperative CT and PET/CT, which accords well with current guideline recommendations. These results also indicate an important fact that most AM cases metastasize to regional lymph nodes first and then to distant lesions via lymph and blood flow [13].

Taking the findings together, our study shows that preoperative CT and PET/CT for patients without lymphadenopathy does not provide efficacy beyond the record of baseline status. Principles of treatment (e.g., performing surgery with/without CLND or performing none of them) are unlikely to be changed by the results of CT and PET/CT and we should not delay therapeutic intervention to wait for CT and PET/CT. For patients with lymphadenopathy, we need to perform preoperative CT and PET/CT to determine which is the better choice: radical surgery or systemic therapy (e.g., targeted therapy and immunotherapy). Our data support the current guideline recommendations, by adding information regarding AM. Imaging did not add much unless patients had palpable adenopathy. Routine preoperative CT and PET/CT may not be warranted for all patients with AM.

## Figures and Tables

**Table 1:** Background data of all 109 patients.

Parameters	Number (%)
Age, years	
Mean (median)	66.3 (69)
Range	16–90
Sex	
Male	46 (42.2)
Female	63 (57.8)
Primary site	
Foot	64 (58.7)
Hand	21 (19.3)
Toenail	14 (12.8)
Fingernail	10 (9.2)
Breslow thickness	
Tis	22 (20.2)
T1	25 (22.9)
T2	14 (12.8)
T3	15 (13.8)
T4	33 (30.3)
Ulceration	
Present	33 (30.3)
Absent	76 (69.7)
Lymphadenopathy	
Present	9 (8.3)
Absent	100 (91.7)
Imaging modality	
CT alone	20 (18.3)
CT and PET/CT	89 (81.7)

CT, computed tomography; PET, positron emission tomography.

**Table 2:** Detection of lymph node and distant metastasis in 100 patients without lymphadenopathy.

	**Detection of Lymph Node Metastasis**			
	**Patients**	**Positive in Imaging**	**Metastasis in Histopathology**	**Positive Predictive Value**
CT alone	17	2 (11.8%)	1 (5.9%)	50.0%
CT and PET/CT	83	15 (18.1%)	1 (1.2%)	6.7%
Total	100	17 (17.0%)	2 (2.0%)	11.8%
	**Detection of Distant Metastasis**			
	**Patients**	**Positive in Imaging**	**Metastasis Confirmed during Follow-up**	**Positive Predictive Value**
CT alone	17	1 (5.9%)	0 (0%)	0%
CT and PET/CT	83	3 (3.6%)	0 (0%)	0%
Total	100	4 (4.0%)	0 (0%)	0%

CT, computed tomography; PET, positron emission tomography.

**Table 3:** Breslow thickness (T category) and metastasis detection in 100 patients without lymphadenopathy.

	**Detection of Lymph Node Metastasis**			
	**Patients**	**Positive in Imaging**	**Metastasis in Histopathology**	**Positive Predictive Value**
Tis	22	4 (18.2%)	0 (0%)	0%
T1	24	4 (16.7%)	0 (0%)	0%
T2	14	2 (14.3%)	0 (0%)	0%
T3	13	2 (15.4%)	0 (0%)	0%
T4	27	5 (18.5%)	2 (7.4%)	40.0%
	**Detection of Distant Metastasis**			
	**Patients**	**Positive in Imaging**	**Metastasis Confirmed during Follow-up**	**Positive Predictive Value**
Tis	22	0 (0%)	0 (0%)	-
T1	24	0 (0%)	0 (0%)	-
T2	14	2 (14.3%)	0 (0%)	0%
T3	13	1 (7.7%)	0 (0%)	0%
T4	27	1 (3.7%)	0 (0%)	0%

**Table 4:** Detection of metastasis in nine patients with clinical lymphadenopathy.

Patients	Age	Sex	Primary site	T category	N category	Site of Distant Metastasis
#1	74	Male	Foot	T1a	N3b	-
#2	74	Male	Foot	T3a	N3b	Liver
#3	64	Male	Foot	T3a	N3b	-
#4	74	Male	Toenail	T4a	N2b	-
#5	79	Male	Fingernail	T4a	N1c	-
#6	63	Male	Hand	T4b	N3c	Paraaortic LN
#7	74	Male	Foot	T4b	N3b	Paraaortic LN
#8	69	Male	Foot	T4b	N3c	Lung
#9	76	Female	Hand	T4b	N1b	-

LN, lymph node.

## Data Availability

The data presented in this study are available on request from the corresponding author. The data are not publicly available because of privacy restrictions.
